# A Novel Erinacine S Derivative from *Hericium erinaceus* Overcomes Chemoresistance in Colorectal Cancer Cells by Enhancing TRAIL/TNFR1/DR5 Expression through Histone Acetylation

**DOI:** 10.7150/ijms.119894

**Published:** 2025-10-10

**Authors:** Shui-Yi Tung, Kam-Fai Lee, Yung-Yu Hsieh, Kung-Chuan Cheng, Ko-Chao Lee, Li-Ya Lee, Wan-Ping Chen, Chin-Chu Chen, Chih-Chuan Teng, Meng-Chiao Hsieh, Cheng-Yi Huang, Hsing-Chun Kuo

**Affiliations:** 1 Division of Gastroenterology and Hepatology, Department of Internal Medicine, Chang Gung Memorial Hospital, Chiayi, Taiwan.; 2 College of Medicine, Chang Gung University, Taoyuan, Taiwan.; 3 Department of Pathology, Chang Gung Memorial Hospital, Chiayi, Taiwan.; 4 Division of Colorectal Surgery, Department of Surgery, Chang Gung Memorial Hospital-Kaohsiung Medical Center, Kaohsiung, Taiwan.; 5 College of Medicine, Chang Gung University, Kaohsiung, Taiwan.; 6 Biotech Research Institute, Grape King Bio Ltd., Taoyuan, Taiwan.; 7 Department of Nursing, Division of Basic Medical Sciences, Chang Gung University of Science and Technology, Chiayi, Taiwan.; 8 Research Fellow, Chang Gung Memorial Hospital, Chiayi, Taiwan.; 9 Division of Colon and Rectal Surgery, Department of Surgery, Chang Gung Memorial Hospital, Chiayi, Taiwan.; 10 Center for Drug Research and Development, Chang Gung University of Science and Technology, Taoyuan, Taiwan.; 11 Chronic Diseases and Health Promotion Research Center, Chang Gung University of Science and Technology, Chiayi, Taiwan.

**Keywords:** Erinacine S, Human chemoresistant human colorectal cancer cells, *Hericium erinaceus* mycelium, H3K9K14ac.

## Abstract

*Hericium erinaceus*, renowned for its pharmaceutical potential, is particularly notable for its isolated diterpenoid derivative, erinacine S. Colorectal cancer (CRC) is one of the most prevalent cancers, characterized by CSC that contribute to chemoresistance and sustained tumor growth. While various drugs have been explored, the precise mechanism underlying multifaceted functions of erinacine S in inhibiting chemoresistant human CRC cells remains elusive. By using annexin-V/propidium iodide staining and a Fluo-3 fluorescence staining assay, the cell death and viability in cancer cells and an* in vivo* xenograft mouse model were measured by western blots and an immunohistochemical assay. This study unequivocally demonstrates that erinacine S treatment significantly induces apoptosis and suppresses the aggressiveness of chemoresistant human CRC cells. Erinacine S also exhibits remarkable inhibitory effects on tumor growth in an* in vivo* xenograft mouse model. Immunohistochemical analyses unveiled that erinacine S treatment significantly upregulates the expression of TRAIL, TNFR1, and DR5 proteins while downregulating p-AKT, p-ERK, HIF1α, PCNA, and NFκB levels in the xenograft mouse model of chemoresistant human CRC cells. Erinacine S treatment of HCT-116/FUR cells triggered the activation of extrinsic apoptosis pathways (TRAIL, TNFR1, DR5, and caspase-3) and exerted a time-dependent suppression on the expression of anti-apoptotic molecules like Bcl-2 in intrinsic pathway. The activation of the p-PAK/FAK/p300 pathways was intricately involved in erinacine S-induced transcriptional activation; this was evidenced by histone H3K9K14ac (Acetyl Lys9/Lys14) modifications in the promoters of TRAIL, TNFR1, and DR5. The inactivation of the CXCR4/PI3K/Akt/HIF-1 pathway played a pivotal role in mediating the capacity of erinacine S to inhibit chemoresistant CRC growth while enhancing tumor apoptosis. Thus, erinacine S demonstrates notable inhibitive effects, both *in vitro* and *in vivo*, through the inhibition of invasion, migration, and proliferation in human chemoresistant cell lines, and holds promise as a natural agent for clinical therapy of patients with CRC.

## Introduction

Colorectal cancer (CRC) ranks as one of the leading causes of global cancer-related mortality, second only to lung cancer in Western countries 1. In the United States, approximately 140 thousand individuals are diagnosed with CRC annually, resulting in 50 thousand deaths 2. A concerning increase in CRC cases has also been observed in Taiwan. The primary cause of mortality in CRC is often attributed to cancer cell metastasis to distant sites. Since CRC is frequently asymptomatic in its early stages, about 35% of patients already have metastatic disease at the time of diagnosis. The post-treatment five-year survival rate for patients with metastatic CRC typically ranges between 10% and 20% 3. Surgical excision remains the gold standard for curing CRC, and timely intervention is crucial upon diagnosis. High-risk individuals diagnosed with stage 2 CRC are often recommended for adjuvant chemotherapy, typically employing the fluorouracil and oxaliplatin (FOLFOX) regimen 4. Chemotherapeutic treatment commonly includes 5FU (5-fluorouracil)-based drug combinations. However, the use of partial chemotherapy (FOLFOX) can lead to the development of drug-resistant CRC, promoting tumor invasion and metastasis 5. One recognized contributor to chemotherapeutic resistance and persistent tumor growth is cancer stem cells (CSCs) 6. These chemotherapeutic drug-resistant CRC cells stem from the primary CRC cell subgroup, differing in molecular and phenotypic characteristics. They are variously referred to as tumor-initiating cells, tumor-promoting cells, or more commonly, CSCs (7). CSCs exhibit resistance to drugs, which may be attributed to mechanisms such as drug efflux (involving membrane protein P-glycoprotein or P-gp), autocrine signaling pathways (macrophage migration inhibitory factor/ C-X-C chemokine receptor type 4 (MIF-CXCR4 axis), alterations in metabolism (influencing hypoxia-inducible factor 1 or HIF-1α signaling), and transitions in epithelial to mesenchymal transition (EMT) 8. The current study aims to explore whether novel dietary compounds, such as erinacine S, can induce apoptosis in drug-resistant CRC cells. Additionally, the study investigates the potential effects and associated mechanisms underlying the inhibition of cancer cell invasion and metastasis.

*Hericium erinaceus*, commonly known as Lion's Mane mushroom, is a medicinally valuable edible fungus with a long history of application in East Asian traditional medicine. This fungus, named after its unique white, furry appearance, was historically regarded as a precious ingredient for both culinary and medicinal purposes 9. The earliest written records of its use can be traced back to the Sui Dynasty. The Ming Dynasty's The Ben Cao Gang Mu provided detailed documentation of *H. erinaceus*, describing it as mild in nature with a sweet taste, beneficial for supporting the five visceral organs and aiding digestion 10. According to the Xin Zhong Hua Ben Cao, the whole *H. erinaceus* possesses a sweet taste with neutral properties, capable of supporting the five visceral organs, promoting digestion, nourishing the body, and exhibiting anti-cancer properties 10. A range of therapeutic properties are associated with this mushroom, including anti-hypoglycemic 11, anti-hypolipidemic 12, anti-inflammatory 13, anti-tumor 14, anti-aging 15, and nerve injury-regulating 16 effects. *H. erinaceus* has also demonstrated potential in countering chronic gastritis, gastrointestinal cancer, and esophagus cancer 17. Previously, the antioxidative properties of the methanol extract from mushrooms like *H. erinaceus* have been highlighted, identified as key contributors to this trait 18. Furthermore, *H. erinaceus* extracts are now marketed as food products and dietary supplements in various countries. Examples include "*Hericium erinaceus* Gastric Health Oral Solution" and "Jiangzhong Lion's Mane Sugar-free Crispy Biscuits" in China, "Natural Lion's Mane Mushroom Tincture" in the United Kingdom, and "Real Mushrooms Lion's Mane Mushroom Capsule Supplements" in the United States. These products are recognized for their potential to improve gastrointestinal function and enhance immune system response 19. However, these investigations primarily utilized extracts from the fruiting body of *H. erinaceus* cultivated via solid-state culture 18. In contrast, the current study focuses on *H. erinaceus* mycelium produced through liquid-state fermentation, enriched with erinacine compounds (erinacine A-I and erinacine S) 20. Existing literature has highlighted the potential of *H. erinaceus* extracts from the fruiting body in resisting liver cancer, astrocytoma, gastric cancer, leukemia, and CRC 21,22. Nevertheless, the specific impact of active constituents extracted using alcohol, particularly erinacine S derived from mycelium produced via liquid fermentation, on drug-resistant CRC cells remains an unexplored field of research.

The loss of control over apoptosis is a pivotal factor that enables cancer cells to evade cell death, thereby promoting their prolonged survival and enhancing invasiveness during tumor progression 23. Apoptosis can be initiated via two primary pathways: the intrinsic and extrinsic pathways. The intrinsic pathway involves the activation of caspases, particularly within the mitochondria, leading to apoptosis upon treatment with various natural compounds 24. B-cell lymphoma 2 (Bcl-2) family members, particularly anti-apoptotic ones like Bcl-2 and Bcl-extra-large (Bcl-XL), are characterized by the presence of at least three BH amino acid sequences. In contrast, the extrinsic pathway involves the interaction of death activators with cell surface receptors, resulting in cell death 25. Prominent examples include tumor necrosis factor (TNF)-α/Fas ligand, TNF, and FasL (Apo-1L or CD95L) 26,27. Our study reveals the intricate mechanisms underlying the inhibition of invasive and metastatic capabilities in drug-resistant CRC cells by *H. erinaceus* erinacine S. This inhibition is achieved through the suppression of the macrophage migration inhibitory factor (MIF)/ C-X-C Motif Chemokine Receptor 4 (CXCR4)/ phosphoinositide 3-kinases (PI3K)/ protein kinase B (Akt)/ extracellular signal-regulated kinase (ERK)/ nuclear factor kappa B (NFκB)/HIF-1α) pathway and the reversal of EMT in HCT-116/5-fluorouracil-resistant (5FUR) cancer cells. Erinacine S induces a programmed cell death process in drug-resistant CRC cells, implicating both intrinsic and extrinsic pathways. Furthermore, erinacine S derived from *H. erinaceus* exhibits the ability to inhibit cell-cycle-related proteins, including CDK2, cyclin D1, and cyclin E, thereby causing cell cycle arrest in the G1 phase. Erinacine S, as an active constituent of *H. erinaceus*, exerts its anti-cancer effects by impeding cancer cell growth, promoting apoptosis, and operating through the p300/ focal adhesion kinase (FAK)/ p21-activated kinase (PAK1) pathway-mediated epigenetic modification of histone H3K9K14ac (acetyl Lys9/Lys14) within the promoters of TNF-related apoptosis-inducing ligand (TRAIL), TNF-receptor 1 (TNFR1), and death receptor 5 (DR5). This epigenetic modulation contributes to the regulation of gene expression in the apoptosis pathway by erinacine S.

## Materials and Methods

### Information on antibodies and chemical reagents

The antibodies were purchased from Santa Cruz Biotechnology (Santa Cruz, CA, USA), including mouse monoclonal antibodies against CDK2, Cyclin D1, Cyclin E, Bcl-2, Oct-4, SOX-2, H3 histone H3K9K14ac (Acetyl Lys9/Lys14), proliferating cell-nuclear antigen (PCNA) and β-actin. The antibodies were respectively obtained by Abcam Technology (Abcam) and Cell Signalling Technology, including mouse/rabbit polyclonal antibodies against phospho (p)-ERK1/2 Thr202 Tyr204, AKT Thr308, PAK Thr423, FAK Tyr577/579, HIF1α, CBP/p300 and PI3 Kinase p85 (Tyr458), NFκB p105/50, TRAIL, TNFR1, DR5. Mouse/rabbit polyclonal antibodies against CD44 and CD133 (Abcam, Cambridge, UK). All culture materials were obtained from Gibco (Grand Island, NY, USA). Protease inhibitor cocktails, the p300-Binding Protein inhibitor C646, Fluo-3-pentaacetoxymethyl ester (Fluo 3-am), the membrane-permeant JC-1 dye, FAK inhibitor Y15, or the PAK1 inhibitor IPA-3, NP-40, sodium deoxycholate, 2,7-dichlorodihydrofluorescein diacetate (H2DCFDA) and 3-(4,5-dimethylthiazol-2-yl)-2,5-diphenyltetrazolium bromide (MTT) were purchased from Sigma (St. Louis, MO, USA).

### Fungus material

*H. erinaceus* (BCRC 35669) was procured from the Bioresources Collection and Research Center (BCRC) at the Food Industry Research and Development Institute, located in Hsinchu, Taiwan. The fungus name was confirmed by consulting List (https://catalog.bcrc.firdi.org.tw/BcrcContent?bid=35669; accessed Feb 18, 2025).

### Hericium erinaceus extracts and analysis of erinacine S

**Figure 1:**
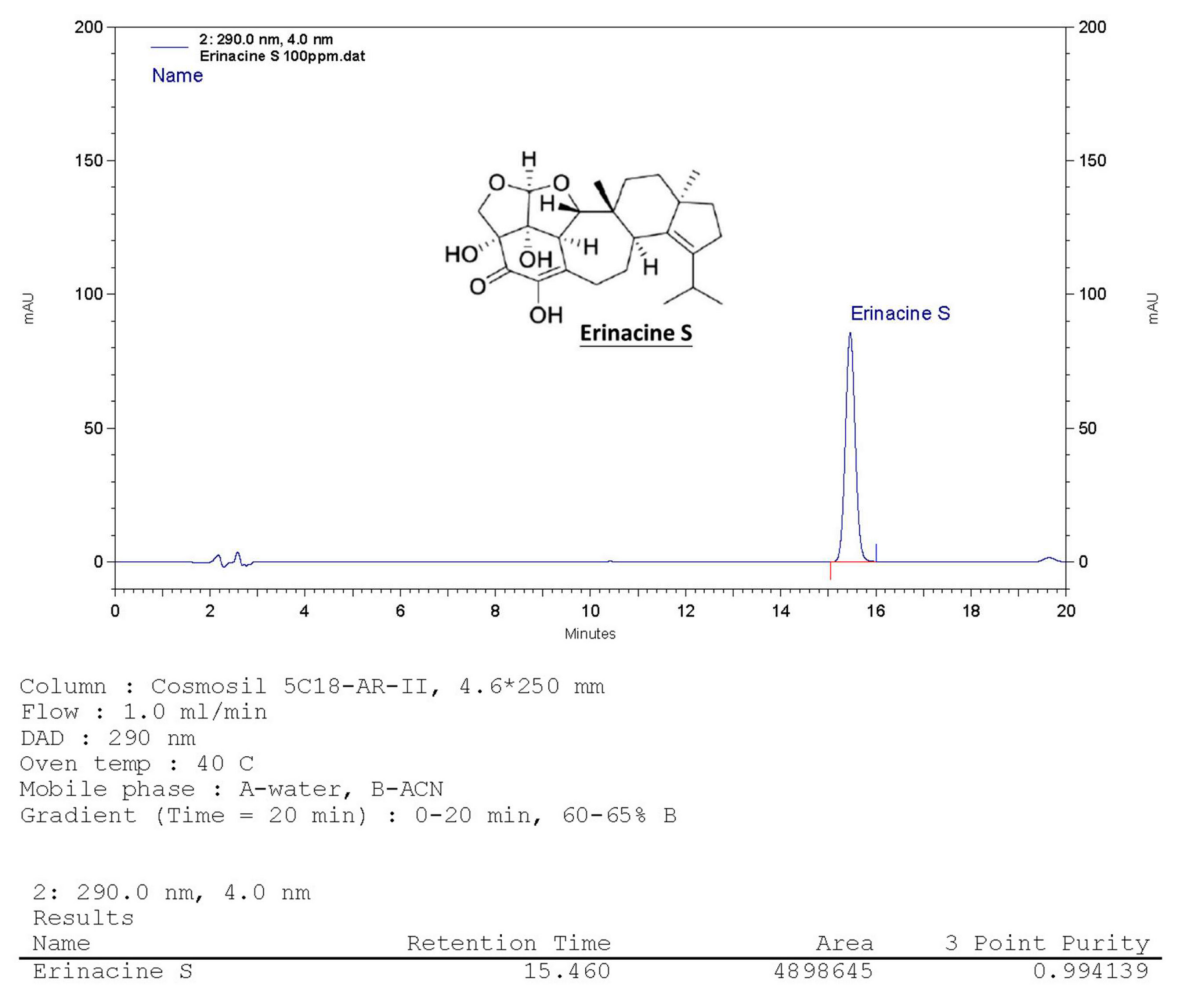
** HPLC analysis of the ethanol *H.**** erinaceus* mycelium extract. The retention time peak at 15.46 min was demonstrated by a bioreactor (UV detection at 290 nm).

The* H. erinaceus* specimen was initially transferred from an agar slant onto a potato dextrose agar plate, where it was then maintained at a temperature of 26°C for a duration of 15 days, consistent with previously established protocols 28. Following the extraction of fresh mycelium from *H. erinaceus* using ethanol, the subsequent fermentation process of the *H. erinaceus* mycelia was carried out. These mycelia were subsequently transformed into a powdered form and subjected to fractionation. Using a COSMOSIL 5C18-AR-II column (250 × 4.6 mm; 5 μm particles; Nacalai USA, Inc.), erinacine S showed a retention time of 15.4 min at 1.0 mL min-1 flow with 290 nm UV detection. The compound's yield from H. erinaceus ethanol extraction was confirmed at ~1 g kg-1 via HPLC 28. Fig. 1 shows erinacine S's chemical structure (PubChem CID: 127047879).

### Development of chemoresistance cell lines

The colon cancer cell line, HCT-116, was procured from the Bioresources Collection and Research Center (BCRC) of the Food Industry Research and Development Institute, based in Hsinchu, Taiwan. These cells were meticulously maintained in Dulbecco's Modified Eagle Medium (DMEM), which was augmented with 10% fetal bovine serum (FBS) and 1% penicillin/streptomycin. This maintenance was carried out within a CO_2_ incubator, preserving the cells at a temperature of 37°C. To establish the 5FU resistant cell line, HCT-116/FUR, a previously established protocol 8 was followed. Initially, parental HCT-116 cells were exposed to an initial dose of 0.1 μg/mL of 5FU, and the surviving cells were cultured until they reached a confluence of 80% over the course of three passages (approximately 6 weeks). Subsequently, the cells that endured this initial 5FU treatment were subjected to a concentration of 0.5 μg/mL of 5FU for another three passages (around 6 weeks), and then further escalated to 1.0 μg/mL for an additional three passages (roughly 6 weeks). Ultimately, the 5FU concentration was raised to 2.0 μg/mL for the subsequent 3 weeks (totaling 10 weeks). These cells, now displaying resistance to chemotherapy, were designated as HCT-116/FUR and continued to be cultured in DMEM supplemented with 5FU 2.0 μg/mL, as HCT-116/FUR chemoresistant cells, 10% fetal bovine serum (FBS), along with penicillin-streptomycin 29.

### Cell cycle distribution analysis and flow cytometry

HCT-116/FUR cells were subjected to treatment with either 0.1% DMSO (used as a control) or erinacine S for a duration of 24 hours. Subsequently, only the surviving cell fraction was collected for measurement through cell cycle distribution analysis, as described in a previous publication 30.

### Measurement of apoptosis assay

Co-staining with Annexin V-FITC and propidium iodide (Biosource International, USA) was employed for the measurement of cell apoptosis, following established procedures. Subsequent to staining, FACS analysis was conducted using the Attune NxT Flow Cytometer (Attune NxT Flow Cytometer, Thermo Fisher Scientific Inc.), as described in a previous publication 31.

### Protein extraction and immunoblot analyses

Cellular lysates were prepared by suspending tumor tissue in lysis buffer. The cellular material was then fragmented through ultrasonic disruption, followed by extraction procedures performed on the generated lysates. The protein content in the supernatant was quantified using a suitable method. For immunoblotting, Immobilon-P membranes (Millipore, Bedford, MA) were employed, along with the appropriate secondary antibodies, following established protocols 32.

### Animal study

The animal care and experimental procedures adhered to in this study were sanctioned by the Institutional Animal Care and Use Committee of Chang Gung Memorial Hospital, Chiayi, Animal Ethics Research Board (IACUC approval: 2020121810). Male BALB/c-nu nude mice, aged 4-6 weeks, with weights ranging from 18 to 20 grams, were procured from the National Laboratory Animal Center in Taiwan. These mice were housed in a specific pathogen-free (SPF) environment and provided with sterilized food and water. For the subcutaneous tumor model, 10^6^ cells of both HCT-116 and HCT-116/FUR cell lines were injected into the flanks of 4-6-week-old male athymic BALB/c-nu mice. Post-tumor implantation, mice were distributed randomly into four experimental groups with six animals per group (n = 6). The HCT-116 control group received daily intraperitoneal administration of 0.1 mL DMSO (0.25%). The experimental groups were administered erinacine S intraperitoneally at doses of 1 mg/day and 5 mg/day, respectively, over a 5-day period. Tumor dimensions were systematically recorded every 4 days using caliper measurements. Additionally, weekly body weight assessments were conducted to monitor for potential toxicity effects of the treatment. Upon completion of the 18-day treatment regimen, the animals were ethically sacrificed, followed by collection of tumors and essential organs (liver, lungs, and kidneys) in accordance with previously established protocols 33.

### Matrigel Invasion and Scratch Assays

A straight wound line was created in the monolayer of the transfected cells, and images of the wound line were subject to analysis using the Openlab v3.0.2 image analysis software (Improvision, Coventry, UK). Matrigel invasion of tumor cells was assessed using the Boyden chamber, following established procedures with a previously described protocol 34.

### Histochemistry and immunohistochemistry analysis

Tumor tissue sections were fixed in 4% formaldehyde and subsequently embedded in paraffin blocks. After hematoxylin and eosin staining, the tissue slides were prepared for microscopic examination. For immunohistochemical analysis, 5-μm-thick sections of each subcutaneous tumor specimen were incubated with monoclonal anti-p-AKT and p-ERK, HIF1α, PCNA, and NFκB p105/50 antibodies (Santa Cruz, CA, USA). Digital images were captured using a digital camera (Canon A640), and the positive area and optical density (OD) of the immunoreactive cells (brown) were analyzed in three randomly selected microscopic fields for each slide. The IHC index was defined as the average integral optical density (AIOD; positive area × OD/total area), following established protocols 35.

### Chromatin immunoprecipitation (ChIP) analysis

HCT-116/FUR cells underwent treatment with 1% formaldehyde to establish DNA-protein crosslinks and were subsequently rotated with antibodies specifically targeting histone H3K9K14ac, alongside 2μl of non-immunized rabbit IgG serving as a negative control. Following elution procedures, DNA fragments were isolated and purified utilizing a ChIP DNA Clean & Concentrator Kit (Zymo), after which quantitative PCR analysis was conducted to amplify the promoter regions of TRAIL, TNFR1, and DR5 genes employing specific primer sequences (Table 1). The data were expressed as a percentage relative to a reference gene, as described previously. The reference gene was used to calculate the percent input for each experiment. The results were statistically analyzed using Student's paired t test. A p-value of <0.05 was considered to be statistically significant 28,33,36,37.

### Statistical analysis

All data, presented as the mean ± standard deviation, were subjected to group comparisons using either Student's t-test or one-way Analysis of Variance (ANOVA), followed by Tukey's Multiple Comparison Test. A statistically significant difference between values was defined at *p* < 0.05, following the methodology outlined by Lee et al. 2023 38.

## Results

### Establishment of chemoresistant HCT-116/ 5-fluorouracil-resistant cell lines

**Figure 2:**
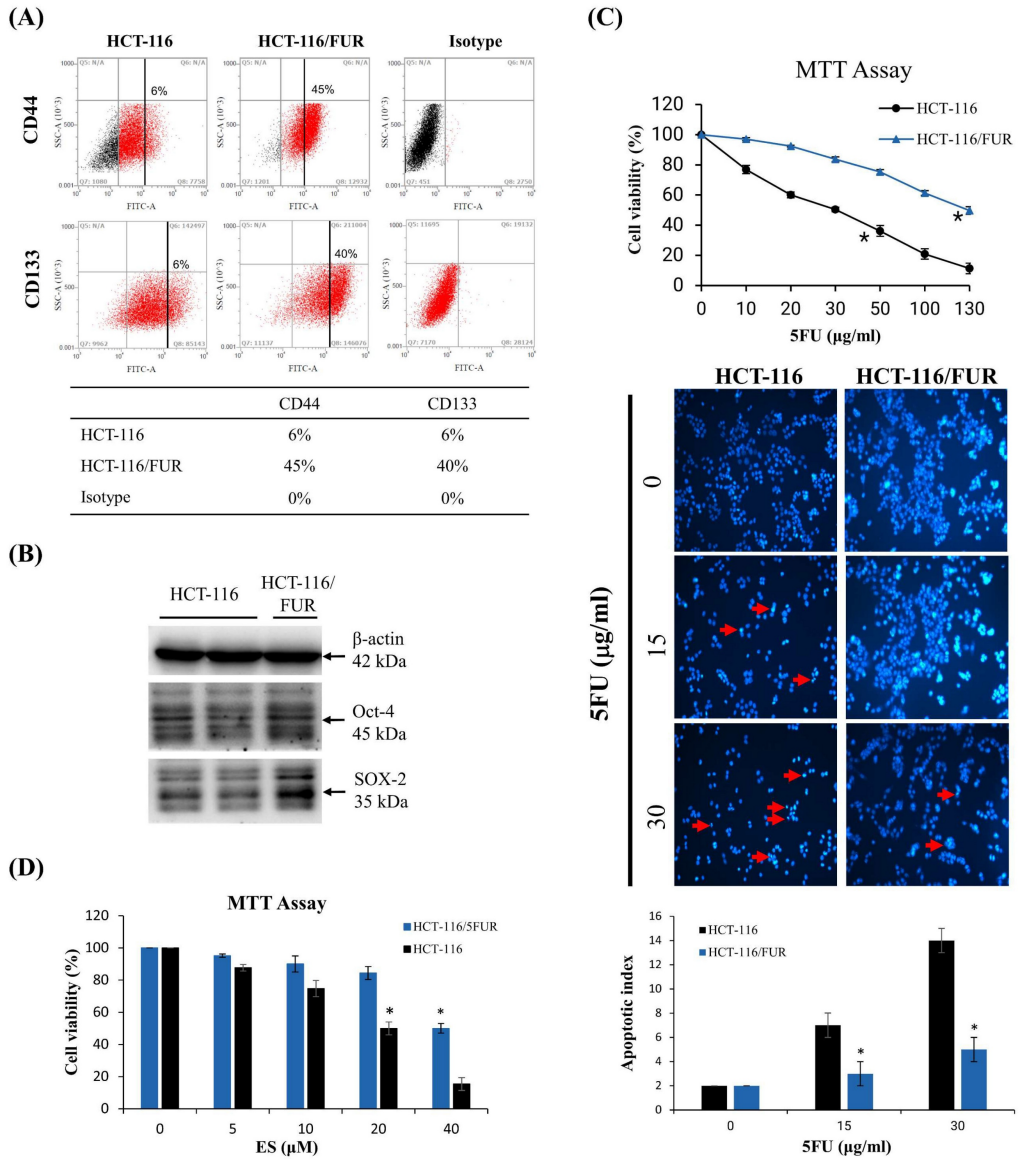
** Chemoresistant cell lines enriched for tumor stem cell markers. (A)** Flow cytometric analysis demonstrating enrichment of chemoresistant cell lines HCT-116/FUR for cells expressing CD133 and CD44 compared with the parental HCT-116 human colorectal cancer cell line. Staining controls include cytometric analysis plots using isotype control antibodies. **(B)** Western blotting showing enriched Oct-4 and SOX-2 proteins in the HCT-116/FUR chemoresistant cells compared with parental HCT-116. Data obtained from western blot are derived from a representative study. **(C, D)** The cell viability was measured through MTT Assay experiments. HCT-116 and HCT-116/FUR were treated with different concentrations of 5FU or erinacine S. *p < 0.05, compared with the HCT-116/FUR group for 24 h.

A small subset of CSCs within cancer cell populations can often elude the effects of chemotherapeutic drugs, subsequently acting as a source of tumor recurrence. Consequently, this study employed a methodology similar to that described by Lee M. Ellis 8,29 to systematically isolate highly drug-resistant CRC cell lines from the initially chemosensitive CRC cell line, HCT-116. Intriguingly, the chemoresistant cell lines exhibited a significantly higher presence of cells expressing CD44 and CD133 compared to their parental counterparts (Fig. 2A). While merely 6% of parental cells demonstrated expression of CD44 and CD133, HCT-116/FUR cells exhibited a notably higher expression of these markers. Moreover, HCT-116/FUR cells displayed a substantially greater expression of Octamer-binding transcription factor 4 (Oct-4) and SRY (sex determining region Y)-box 2 (SOX-2), which are markers associated with putative CSC-like cells 7,8, compared to the parental HCT-116 human CRC cells (Fig. 2B). These findings underscore the presence of CRC cells with enhanced survival capabilities and anti-apoptotic activity, characteristics commonly associated with CRCs 29. The MTT assay was conducted to compare the cytotoxicity of 5-fluorouracil (5FU) in two cell lines: HCT-116 cells and their resistant variant, HCT-116/FUR cells. The IC_50_ (concentration that inhibits 50% of cell growth) for 5FU was 30 μg/ml in HCT-116 cells and 135 μg/ml in HCT-116/FUR cells. The resistance in HCT-116/FUR cells was induced through continuous culture with an unknown concentration of 5FU (Fig. 2C). And then treatment with 5FU at the IC_50_ concentration induced apoptotic cell death in both HCT-116 and HCT-116/FUR cells, as confirmed by DAPI staining. Specifically, the treatment with 30 μg/ml 5FU resulted in an apoptotic index of 14 for HCT-116 cells compared to 5 for HCT-116/FUR cells (Fig. 2C). Additionally, after treatment with 40 μM ES (IC_50_ in HCT-116/FUR), the cell viability had an index of 20% for HCT-116 cells and 50% for HCT-116/FUR cells (Fig. 2D).

### In vitro effect of erinacine S-inhibited migration and invasion as well as tumor growth of chemoresistant HCT-116/FUR cells

**Figure 3:**
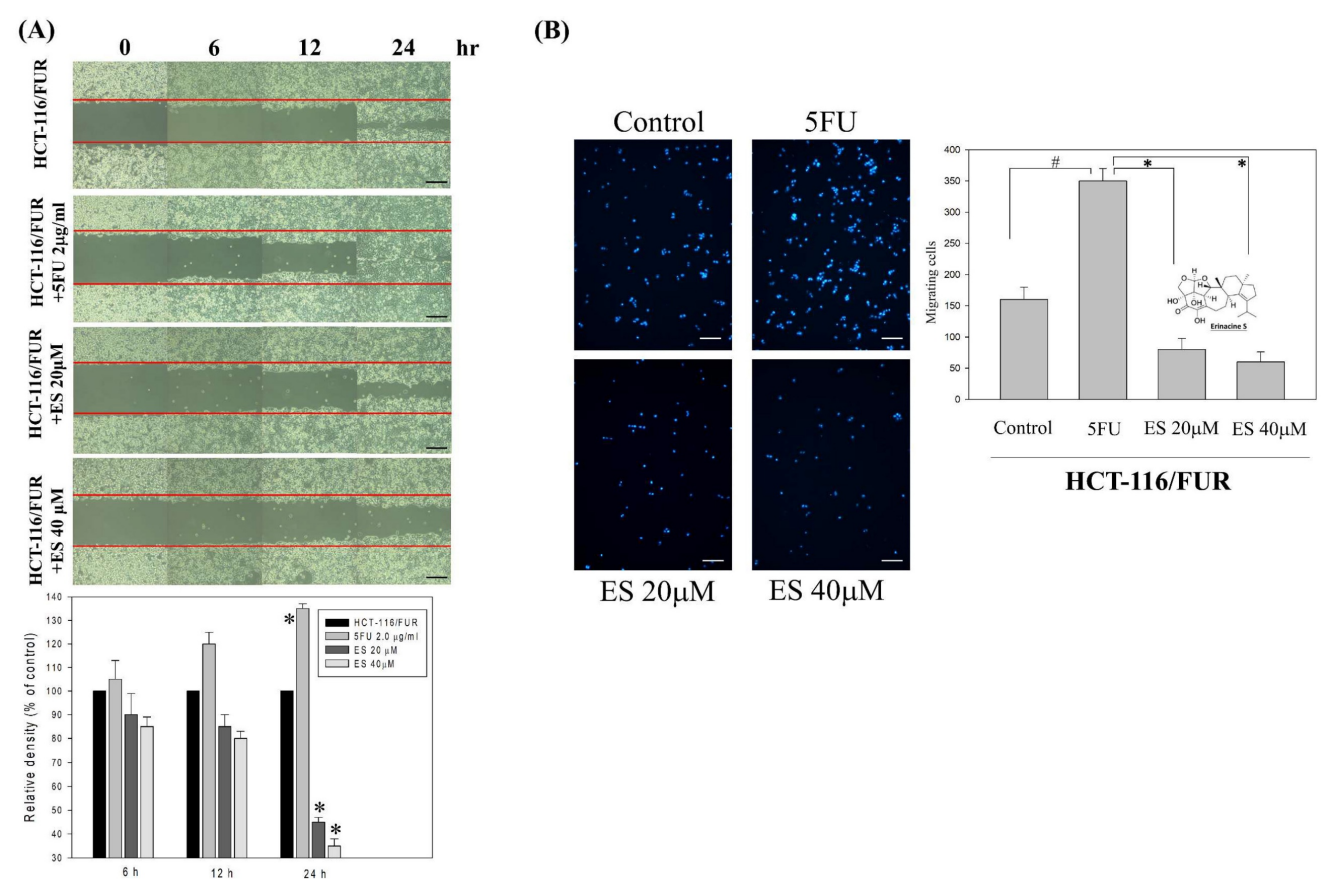
** Effects of erinacine S on *in vitro* cell migration and invasiveness of human HCT-116/FUR chemoresistant cells. (A)** HCT-116/FUR chemoresistant cells were incubated with erinacines S for 12, 24 and 48 h, and the migration was visualized using the scratch-wound assay as described in the 'Materials and methods section'. The percentage of surface area filled by the HCT-116/FUR cells was subsequently quantified through densitometric analyses relative to the control, set at 100% in the graph. Data are presented as means ± standard deviation (SD) based on three independent experiments. The experiments were performed in triplicate, and data are presented as means ± SD. *p < 0.05, compared with the control group for 24 h. **(B)** Effect of erinacine S on the invasive ability of HCT-116/FUR cells. Cells were incubated with various concentrations of erinacine S for 24 h. Invasion through a layer of Matrigel was determined by the Boyden Chamber method as described in 'Materials and methods'. The lower and upper chemotaxis cells were separated by a polycarbonate membrane. Microscopic images detecting cells that migrated into the inner membrane, magnification: 200 ×. The cell migration was quantified by counting the number of cells migrating into the inner membrane. Control cells were untreated. The experiments were performed in triplicate, and data are presented as means SD. The symbol * indicates significantly different means when compared to the control group. *p < 0.05, compared with the 5FU treated group for 24 h.

**Figure 4:**
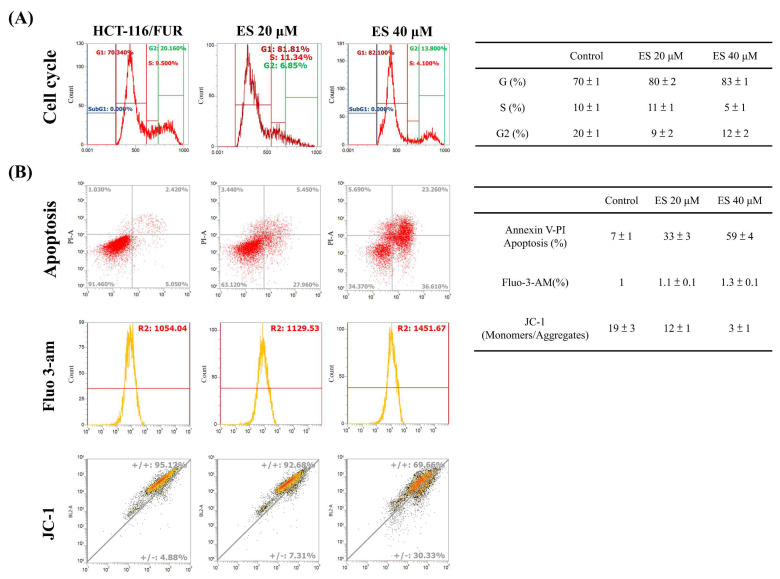
** Effect of erinacine S on cell cycle and apoptosis distribution in HCT-116/FUR chemoresistant cells. (A)** After treatment with Erinacine S for 24 h, the HCT-116/FUR cells were fixed and stained with propidium iodide, and the DNA content was analyzed by flow cytometry (FACS). The percentage of cells in each phase (G1, S, and G2/M) of cell cycle was calculated and expressed. **(B)** After the indicated treatment for 24 h, the HCT-116/FUR cells were stained with fluorescence isothiocyanate-conjugated Annexin-V and propidium iodide for flow cytometry analysis as described in 'Materials and methods'. The percentages presented in each frame depict the apoptotic cells.

For the extraction and purification of erinacine S from fresh mycelium of *H. erinaceus* using ethanol, previously established protocols were followed 28. To evaluate the inhibitory effect of erinacine S on the growth of chemoresistant HCT-116/FUR cells, various concentrations were administered. First, the scratch-wound assay demonstrated that in comparison to the untreated group (HCT-116/FUR) for 24 h, the HCT-116/FUR cells exhibited enhanced migration more aggressively in response to upregulation due to 5FU treatment (Fig. 3A). However, in the presence of erinacine S, migration was significantly inhibited after 12 and 24 h, and resulted in nearly complete inhibition of cell migration when using 40 μM erinacine S (Fig. 3A, *p < 0.05). Furthermore, the Boyden chamber assay revealed more than 23% and 15% reduction in cell invasion when treated with 20 μM and 40 μM erinacine S, respectively, compared to the chemoresistant HCT-116/FUR treated 5FU group (*p < 0.05, Fig. 3B). Flow cytometry analysis (Fig. 4) indicated that a higher proportion of HCT-116/FUR cells treated with erinacine S were arrested in the G1 stage of the cell cycle (G1 arrest), compared to the chemoresistant HCT-116/FUR, showed as percentages of 70%, 80%, and 83% (Fig. 4). Apoptosis analysis using Annexin-V and PI staining also revealed a significant dose-dependent increase in apoptotic induction (7%, 33%, and 59%) by erinacine S in HCT-116/FUR cells (Fig. 4). The cells displayed altered spatial dynamics of extracellular Ca2^+^ signaling (1-fold, 1.1-fold, and 1.3-fold) and demonstrated depolarization of mitochondrial potential (ΔΨm), as indicated by the decrease in the ratio of fluorescence intensities of monomers green /aggregates red 3,13,21, compared to the control group without erinacine S treatment, and observed through flow cytometry experiments (Fig. 4).

### *In vivo* growth inhibition of chemoresistant HCT-116/FUR cell xenograft by erinacine S treatment

**Figure 5:**
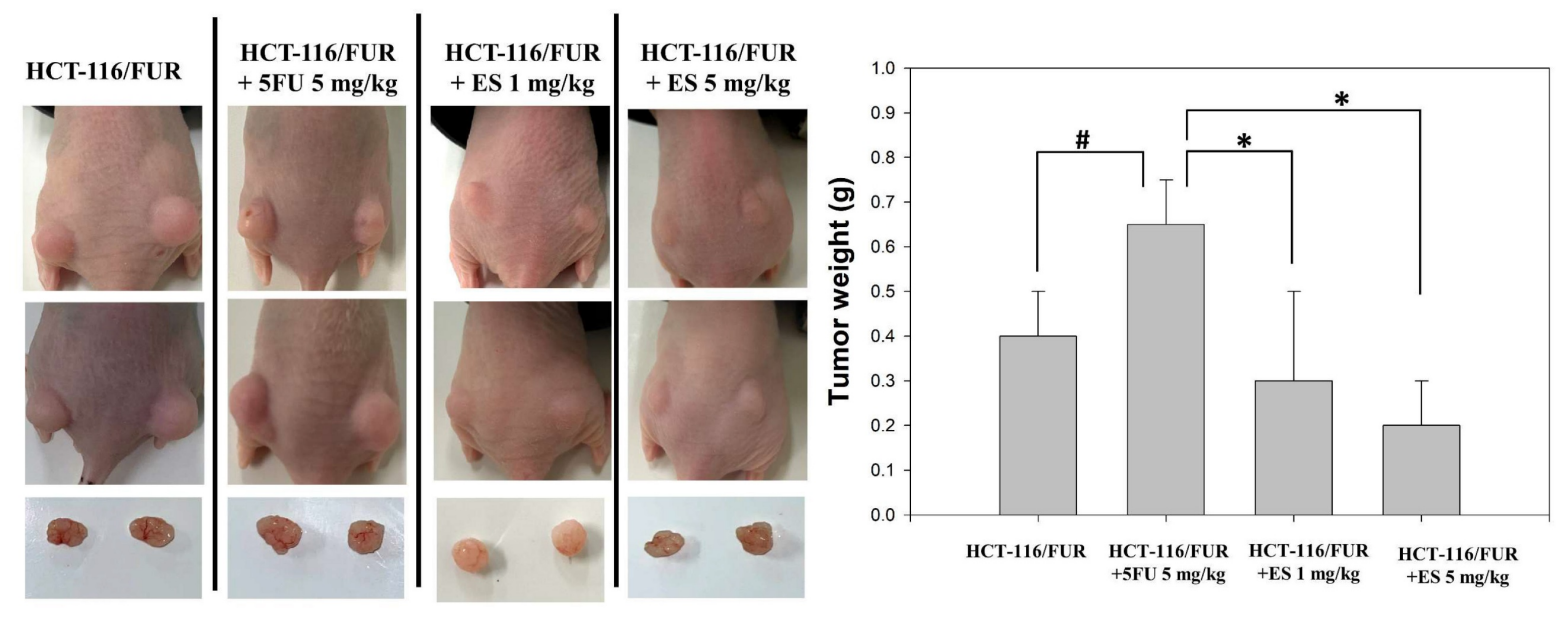
** Growth inhibition of HCT-116/FUR chemoresistant cells xenograft due to erinacine S treatment.** Nude mice were subcutaneously implanted with HCT-116/FUR into flanks on day 0 and treated with or without (as a control) erinacine S as described in the “Materials and methods.” Time course effect of erinacine S on the growth of HCT-116/FUR cells xenograft evaluated in terms of the tumor volume every at 18 days. ^#^*p* < 0.05, as compared to the HCT-116/FUR untreated group; *p < 0.05, as compared to the HCT-116/FUR 5FU 5mg/kg treated group.

**Figure 6:**
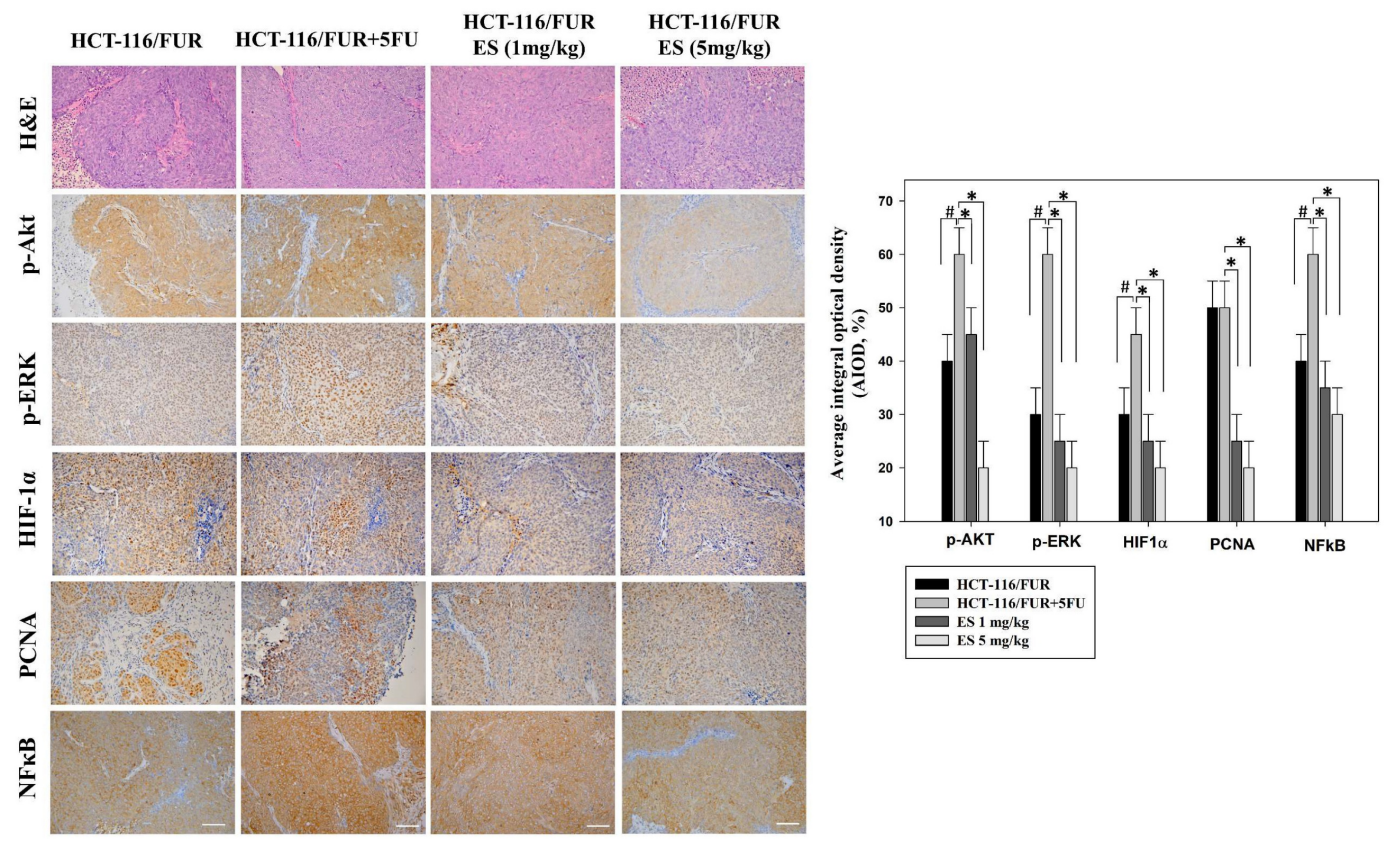
** Alteration in the level of proliferative and apoptotic proteins associated with HCT-116/FUR chemoresistant cells in the *in vivo* xenograft mouse model after erinacines S treatment.** The tumors of the nude mice, with the indicated treatment, were harvested and prepared for hematoxylin and eosin staining (1st row, upper panel) and the measurement of protein levels of phosphorylated protein kinase B (p-AKT; 2nd row, upper panel), p300 (3rd row, upper panel), hypoxia inducible factor 1a (HIF1α; 4th row, upper panel), proliferating cell nuclear antigen (PCNA; 5th row, upper panel), and nuclear factor kappa B (NF-κB1 p105/p50; 6th row, upper panel), by immunohistochemical analysis. Bottom panel: Quantitative immunohistochemical proteins, p-AKT, p-ERK, HIF1α, PCNA, and NFκB stain, were evaluated by calculating the Average of Integrated Optical Density (AIOD). Per treatment group, multiple tumor fields were evaluated. The positive stained area was examined from three randomly-selected observational fields of each section. The data are expressed as mean ± standard deviation. (n = 6/group). ^#^ p < 0.05, as compared to the HCT-116/FUR untreated group; *p < 0.05, as compared to the HCT-116/FUR 5FU 5mg/kg treated group, at a magnification of ×200.

The *in vitro* findings regarding the anti-chemoresistant effects of erinacine S were corroborated by employing an *in vivo* nude mouse model utilizing chemoresistant HCT-116/FUR cell xenografts. Time-course analysis unveiled a significant reduction in tumor weight of the HCT-116/FUR cell xenografts in nude mice upon erinacine S treatment in a dose-dependent manner at 18 days (Fig. 5). These results indicated the HCT-116/FUR xenograft with 5FU 5mg/kg volumes were increased 63% more than HCT-116/FUR xenograft untreatment group (^#^*p* < 0.05), which was shown HCT-116/FUR with 5FU group aggress more better. Under the same HCT-116/FUR xenograft treated with 5FU 5mg/kg, a clear inhibitory effect of erinacine S on the growth of chemoresistant HCT-116/FUR cell tumors, with 46% and 30% reductions compared to the HCT-116/FUR 5FU treated group (*p < 0.05, Fig. 5). Furthermore, the tumors were examined upon removal and quantified through tissue staining, including hematoxylin and eosin staining, and p-AKT, p-ERK, HIF-1α, proliferating cell nuclear antigen (PCNA), and NFκB p50 staining. The levels of p-AKT (a marker associated with the aggressive status of chemoresistant cells), p-ERK (related to a signaling pathway associated with chemosensitivity), HIF-1α (a key regulator implicated in drug resistance), PCNA (a marker for cell proliferation), and NFκB p50 (a marker for the transduction of proliferative signals) were measured in these experimental animals. The results displayed the HCT-116/FUR xenograft with 5FU 5mg/kg volumes were increased expression of p-AKT, p-ERK, HIF-1α, and NFκB p50 than HCT-116/FUR xenograft untreatment (^#^*p* < 0.05, Fig. 6). In addition, erinacine S treatment caused a reduction in the expression of p-AKT, p-ERK, HIF1α, PCNA, and NFκB, compared to the HCT-116/FUR 5FU treated group without erinacine S treatment (*p < 0.05, Fig. 6). The treatment with erinacine S is clearly in accordance with the *in vitro* data.

### Erinacine S-mediated activation of the extrinsic cell apoptosis pathway in chemoresistant HCT-116/FUR cells

**Figure 7:**
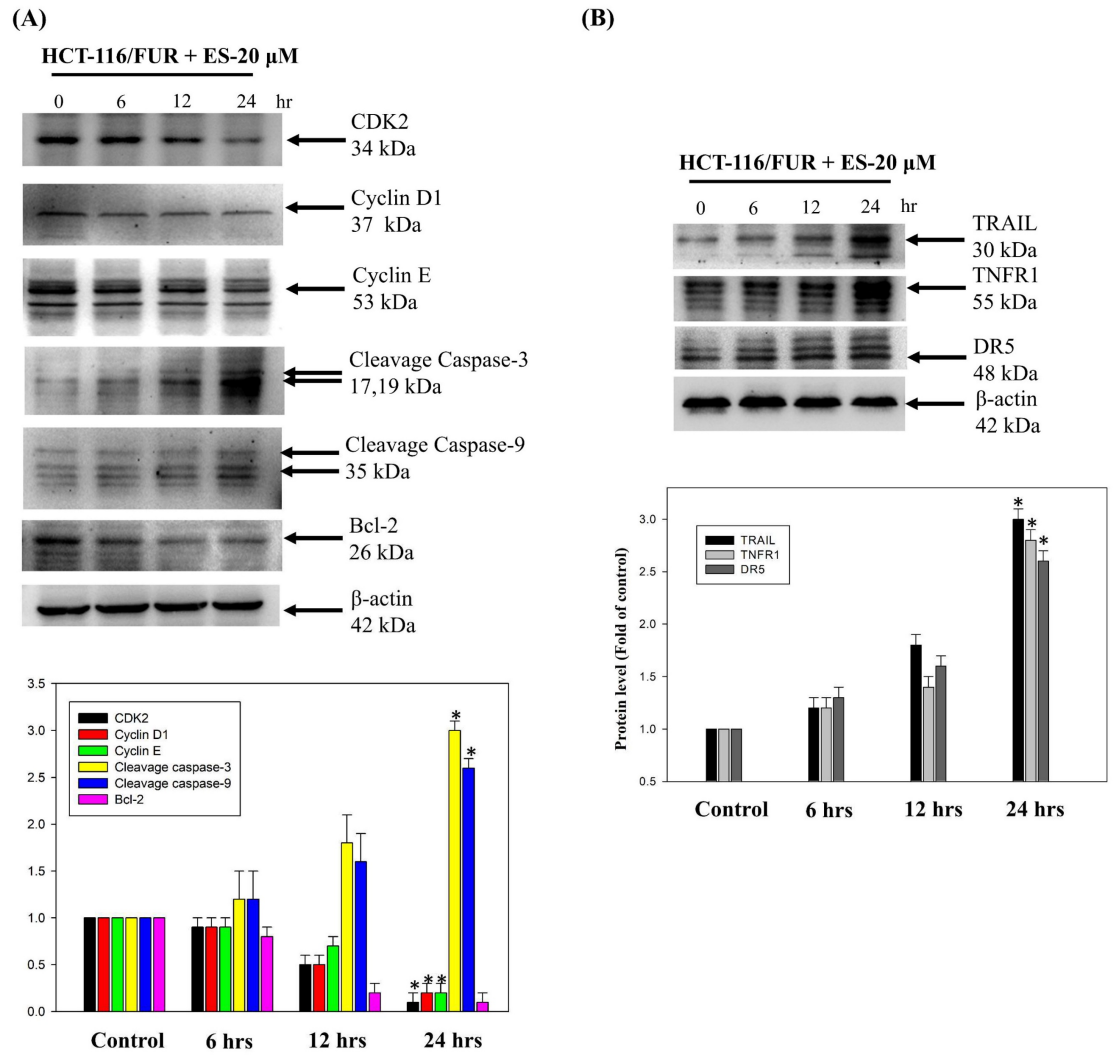
** Effect of erinacines S on the protein level of the molecules-related to the cell cycle and cell death in HCT-116/FUR chemoresistant cells.** The protein level of proteins (including CDK2, cyclin D1, cyclin E, cleavage caspase 3, cleavage caspase 9, Bcl-2, and TRAIL, TNFR1 and DR5) in erinacines S (ES)-treated HCT-116/FUR cell for 0-24 h were detected by western blotting (B). Here, β-actin served as internal control. *p < 0.05, as compared to the HCT-116/FUR untreated group.

To further assess the apoptotic mechanisms induced by erinacine S in chemoresistant HCT-116/FUR cells, we examined cell-death and growth-related proteins. Specifically, we measured expressions of CDK2, cyclin D1, cyclin E, cleaved caspase 3, cleaved caspase 9, Bcl-2, TRAIL, TNFR1, and DR5 by western blotting in erinacine S-treated HCT-116/FUR cells. Thus, erinacine S treatment led to an increase in the active forms of caspase-3 and -9, along with enhanced expression of the upstream proteins TRAIL, TNFR1, and DR5 (Fig. 7A and 7B). These proteins are widely recognized as hallmarks of apoptotic cell death. In contrast, following erinacine S treatment, the levels of CDK2, cyclin D1, cyclin E, and the anti-apoptotic protein Bcl-2 decreased in HCT-116/FUR cells (Fig. 7A). Our findings suggest a novel biological property of erinacine S, inducing apoptotic cell death and cell cycle arrest in chemoresistant HCT-116/FUR cells. This effect is achieved through the regulation of a complex protein network involved in extrinsic cell apoptosis and cell cycle pathways.

### Modulation of C-X-C chemokine receptor type 4-related signaling pathway by erinacine S in chemoresistant HCT-116/FUR cells

**Figure 8:**
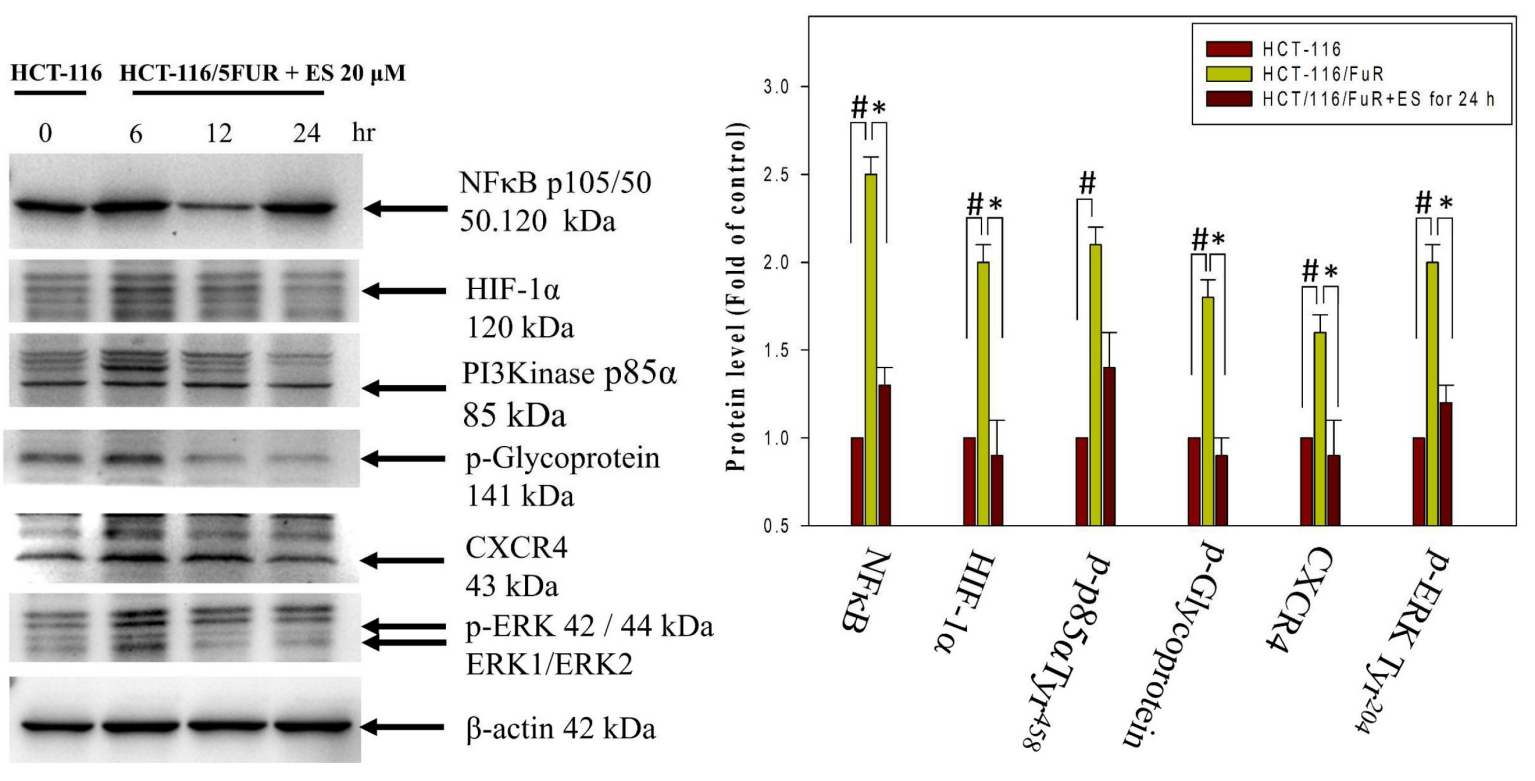
** Inactivation of the CXCR4/PI3K/ERK and NFκB/HIF1α/p-GP pathways by erinacine S treatment.** Western blotting to determine the protein levels of phosphorylated phosphoinositide 3-kinases (PI3K), extracellular signal-regulated kinase (ERK), and β-actin in the HCT-116/FUR chemoresistant cells treated with or without erinacine S at indicated times. ^#^*p* < 0.05, as compared to the HCT-116 untreated group; *p < 0.05, as compared to the HCT-116/FUR untreated group.

Chemoresistant cells often develop resistance through various mechanisms, including increased drug efflux (mediated by membrane protein P-gp), activation of autocrine signaling pathways (such as the MIF-CXCR4 axis), metabolic changes (involving HIF-1α), and alterations in the EMT process. These changes contribute to the aggressiveness of chemoresistant cells and are linked to the CXCR4-MIF axis/PI3K/Akt/HIF-1 pathways. We observed a significant increase in CXCR4 and HIF1α expression, as well as the phosphorylation of PI3K and ERK1/2, and NFκB p50 in HCT-116/5FUR cells compared to human parental HCT-116 cells. The latter are often used as a model for studying cell signaling and apoptosis in human CRC cells (Fig. 8). Furthermore, assessment of the effects of erinacine S treatment of HCT-116/FUR cells in terms of the pathways indicated a time-dependent inhibition of the CXCR4/PI3K/Akt/ERK/HIF-1α/NFκBp50 pathways, resulting in reduced cell proliferation and enhanced apoptosis (Fig. 8). Thus, erinacine S may exert its anti-cancer effects by targeting these critical signaling pathways associated with chemoresistance and cell aggressiveness.

### Erinacine S increases the expression of TRAIL, TNFR1, and DR5 via PAK/FAK/p300-mediated epigenetic histone acetylation in chemoresistant HCT-116/FUR cells

**Figure 9:**
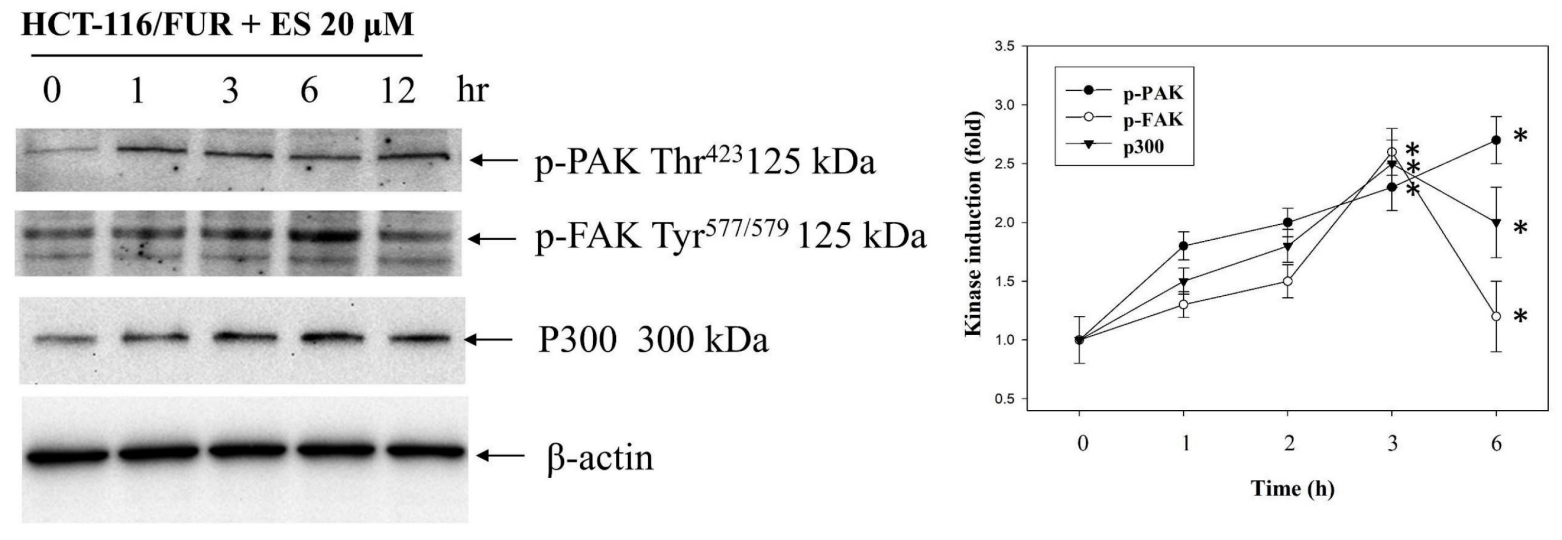
** The effect of erinacine S (ES) on PAK/FAK/p300 pathways in HCT-116/FUR cells.** The protein levels of phosphorylated p21-activated kinase (PAK), focal adhesion kinase (FAK), and histone acetyltransferase p300 in the HCT-116/FUR cells treated with or without erinacine S were determined at the indicated time by western blotting. The protein levels were quantified by densitometry with the ratio of the untreated control set as 1-fold. The quantitative data are presented as the mean of three repeats from three independent experiments. *p < 0.05, compared with the HCT-116/FUR untreated group.

**Figure 10:**
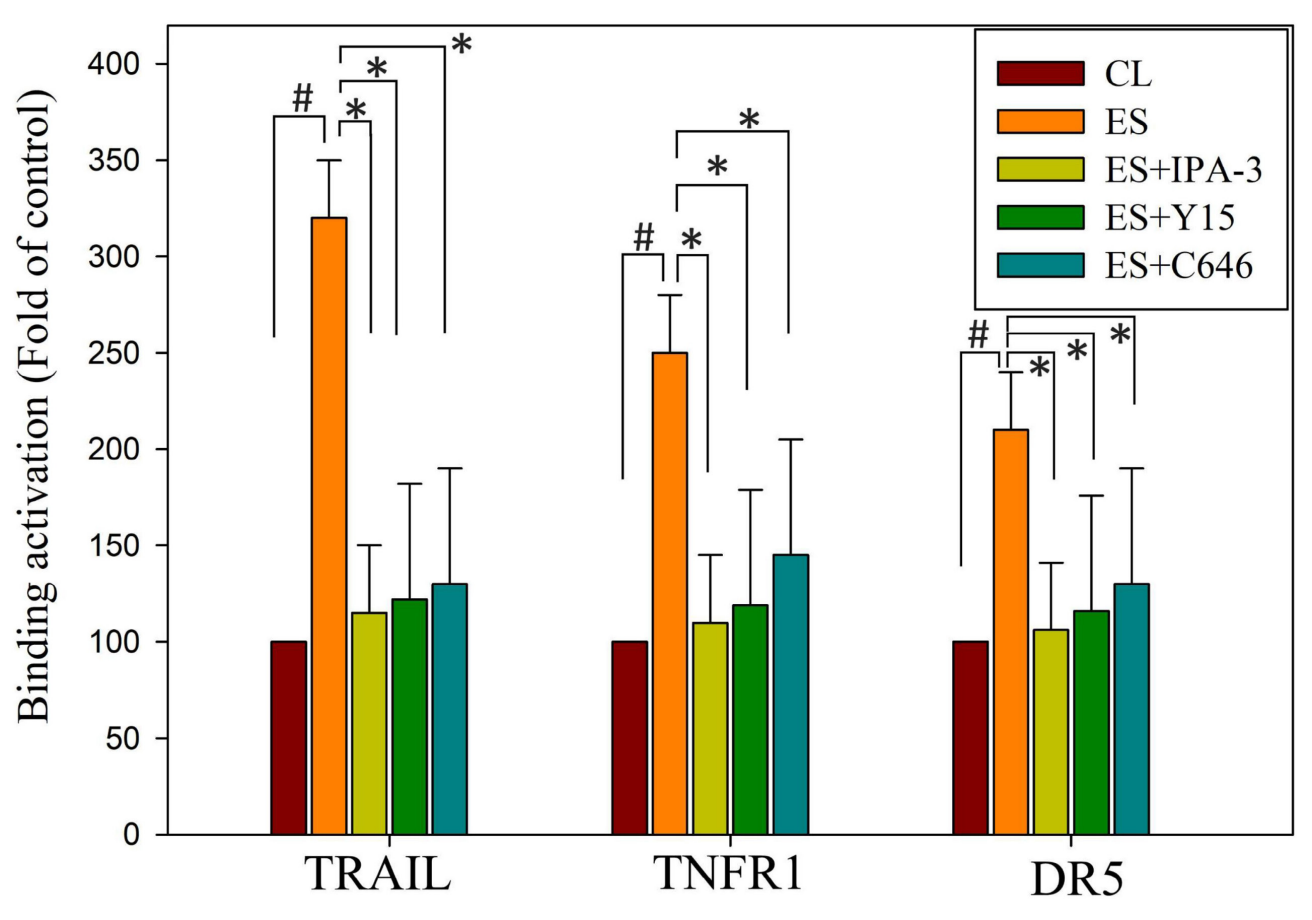
** Erinacine S-mediated epigenetic histone H3K9K14ac (Acetyl Lys9/Lys14) of tumor necrosis factor-related apoptosis-inducing ligand (TRAIL), TNFα1, and death receptor 5 (DR5) promoter involves the reactive oxygen species (ROS)-derived and p21-activated kinase (PAK)/focal adhesion kinase (FAK)/P300 signals.** In this study, HCT-116/FUR cells were treated with erinacine S with or without specific inhibitors for PAK1 (IPA-3), FAK (Y15), or p300 (C646) for 24 h. Chromatin immunoprecipitation (ChIP) assays were performed using antibodies against histone H3K9K14ac, and the precipitated DNA was quantified by real-time polymerase chain reaction targeting TRAIL, TNFα1, and DR5 promoters. The anti-IgG antibody served as a control, and input DNA was amplified for normalization. Normalized mean CT values were calculated as ΔCT, with ΔΔCt used to determine the effect of erinacine S treatment on specific genes. The results, presented as mean ± standard deviation of three independent experiments with triplicate technical repeats, revealed significant differences. ^#^*p* < 0.05, compared to that of the HCT-116/FUR untreated control chemoresistant cells; *p < 0.05, as compared to the HCT-116/FUR erinacine S treated group.

Our study delved into the mechanisms through which erinacine S enhances the expression of death activators TRAIL, TNFR1, and DR5 in chemoresistant cells through acetylation of histone H3 (H3K9K14ac). This histone modification is known to be associated with the upregulation of these genes 39. We explored several signaling pathways activated by erinacine S, including ERK1/2, c-Jun kinase (JNKs), protein 38 (P38), and other kinases like PAK (p21-activated kinase), FAK, and p300. Erinacine S treatment led to the phosphorylation of PAK Thr423, activation of FAK Tyr577/579, and sustained expression of p300 in HCT-116/FUR cells with time (Fig. 9). These events are crucial for the induction of cell death activators through signaling pathways and the epigenetic modification of histone H3K9K14ac binding to the promoter regions of TRAIL, TNFR1, and DR5 genes. In our chromatin immunoprecipitation (ChIP) assay, we found that erinacine S treatment increased the H3K9K14ac modification of histone H3 on the promoter regions of TRAIL, TNFR1, and DR5 genes. These modifications were reversed through the inhibition of multiple protein kinases, as the specific PAK1 inhibitor IPA-3, FAK inhibitor Y15, or p300 inhibitor C646 (Fig. 10). These outcomes suggest that erinacine S exerts its effects by modifying histones and promoting gene expression associated with cell death. Therefore, as a novel biological property, erinacine S inhibits growth and apoptosis in chemoresistant HCT-116/FUR cells by epigenetic histone H3K9K14ac (Acetyl Lys9/Lys14) modification of TRAIL, TNFR1, and DR5 genes. Indeed, treatment with erinacine S increased the level of p-PAK, p-FAK, and p300. Assessment of the effects of kinase inhibitors in blocking erinacine S -induced cell death, and determination of apoptosis (%) by flow cytometry analysis for Annexin-V and PI revealed that PAK1/FAK/p300-specific inhibitors almost blocked erinacine S-induced cell death by 5%, 7%, 5%, respectively (Table 2). Overall, our results reveal a novel biological property of erinacine S, which affects the growth and apoptosis of chemoresistant HCT-116/FUR cells through epigenetic histone modification (H3K9K14ac) of TRAIL, TNFR1, and DR5 genes. This intricate interplay of signaling pathways and histone modifications contributes to the anti-cancer effects of erinacine S.

## Discussion

Erinacines are a group of bioactive compounds predominantly sourced from *H. erinaceus*, commonly referred to as lion's mane mushroom. These compounds have garnered considerable attention due to their health-promoting properties, including their apoptotic-inducing capacity in cancer cells 40. This apoptotic process, driven by bioactive compounds isolated from *H. erinaceus*, is initiated through the generation of reactive oxygen species (ROS) and subsequent mitochondrial dysfunction, subsequently causing the activation of caspases, and orchestrating a cascade of events that lead to cellular demise 28,32,34,36. However, there exists a notable gap in our understanding regarding the molecular mechanisms underlying the action of erinacine S, a compound isolated from *H. erinaceus* mycelium, particularly in the context of human chemoresistant CRC. Within this study, we embarked on the exploration of molecular changes that transpire in drug-resistant CRC cells following prolonged exposure to anticancer agents 8,28,41. These drug-resistant cells, denoted HCT-116/FUR, were meticulously examined to delineate their metastatic capabilities and the mechanisms at play in drug-resistant CRC cells. Our investigations encompassed the characterization of expression profiles in parental HCT-116 human CRC cells and their 5FU-resistant counterparts, employing methodologies such as fluorescence-activated cell sorting and western blot analysis. Strikingly, in contrast to parental HCT-116 cells, HCT-116/FUR cells exhibited significantly augmented expression of CD44 and CD133, putative CSC markers, alongside the stem cell markers OCT4 and SOX2 (Fig. 2). The observed upregulation of the CXCR4/PI3K/Akt/ERK/HIF-1α/NFκB/p50 pathways in drug-resistant CRC cells significantly hindered apoptosis, elucidating a significant role in chemoresistance (Fig. 8). Importantly, our study unveiled a hitherto unreported function of erinacine S, derived from *H. erinaceus* mycelium, namely its potential to suppress the viability of chemoresistant cells both *in vitro* (HCT-116/5FUR cells) and *in vivo* (xenograft mouse model) (Fig 3 and 5). Furthermore, erinacine S induced cell cycle arrest in HCT-116/FUR cells at the G1 stage (Fig. 4 and 5), and triggered apoptosis in a time-dependent manner, as evidenced by indicators like depolarization of mitochondrial potential (DYm) and elevated intracellular Ca^2+^ levels (Fig. 4). Crucially, intraperitoneal administration of erinacine S (at doses of 1 or 5 mg/kg/day) significantly reduced tumor weight within the HCT-116/FUR xenograft model in nude mice compared to the group treated with 5FU (Fig. 5A). Additionally, erinacine S treatment correlated with decreased expressions of p-AKT, p-ERK, HIF1α, PCNA, and NFκB p50 in the tumor region of the HCT-116/FUR xenograft in nude mice (Fig. 6). Our findings further confirmed the activation of caspase 3 and caspase 9 in HCT-116/FUR cells following treatment with erinacine S. Erinacine S treatment also resulted in the inhibition of the proapoptotic protein Bcl-2, and influenced cell-cycle-related proteins, including CDK2, cyclin D1, and cyclin E, leading to cell cycle arrest at the G1 phase (Fig. 7A and 7B).

Lee M. Ellis has illuminated the complex molecular alterations occurring within chemoresistant colorectal cancer cells, specifically concerning intracellular ATP stability regulation and its effects on HIF-1α, a crucial mediator of drug resistance 42,43. Following Ellis's methodology, we established drug-resistant colorectal cancer cell lines in our investigation of chemoresistance mechanisms, with particular emphasis on HCT-116/FUR chemoresistant colorectal cancer cells 44. During our exploration of the fundamental molecular mechanisms underlying drug resistance in colorectal cancer cells 29, we examined the sophisticated interactions between various factors. Our findings revealed that drug-resistant colorectal cancer cells demonstrated upregulated expression of multiple components including the MIF-CXCR4 axis, Bcl-2/Bcl-XL proteins, and P-glycoprotein 29. This upregulation substantially suppressed cellular apoptosis, primarily through the stimulation of the PI3K/Akt/ERK1/2/NFκB signaling cascade. Our study thus provides valuable insights into the mechanisms by which erinacine S induces apoptosis and G1 cell cycle arrest in HCT-116/FUR cells. These effects are intricately associated with the deactivation of the CXCR4/PI3K/AKT/ERK pathway and the NFκB/HIF1α/P-gp pathway. These findings hold promise in the development of innovative strategies to address chemoresistant CRC (Fig. 8). Mani et al. conducted groundbreaking research involving the use of Snail or Twist transcription factors to induce EMT 45. This process led to the transformation of cancer cells into CSCs with drug-resistant properties, marked by stem cell characteristics and the self-renewal ability 46. Building upon this research, future studies may explore the impact and underlying mechanisms of *H. erinaceus* erinacine S on curtailing the invasive and metastatic potential of drug-resistant CRC cells, particularly HCT-116/FUR cells.

Numerous dietary phytochemicals have attracted substantial attention due to their potential to modulate the mitogen-activated protein kinase pathway and others, including the AKT/FAK/PAK1 pathways 28,47. These compounds have demonstrated the capacity to regulate cellular motility and adhesion, inhibit cancer growth, and trigger tumor apoptosis. The impact of these phytochemicals can be contingent upon factors such as concentration, synergistic interactions with other compounds, and the specific chemoresistant cancer type being examined. Notable examples of these phytochemicals, such as resveratrol, curcumin, epigallocatechin gallate, quercetin, genistein, sulforaphane, and berberine, are naturally abundant in food and nutraceuticals 48-50. They exhibit promising inhibitory effects for both cancer prevention and treatment. In line with our previous investigations, our current research extends to the examination of erinacine A, another compound derived from *H. erinaceus* mycelia. Erinacine A has demonstrated remarkable anticancer properties in human CRC cells. This action is mediated through the ROS-mediated p70S6K/NF-κB/p21 pathway, ultimately resulting in cell cycle arrest and the inhibition of cell proliferation 34. The upstream signals involved in erinacine A action encompass both the PI3K/mTOR/p70S6K and ROCK1/LIMK2/Cofilin pathways 32. Erinacine A has been implicated in the remodeling of the actin cytoskeleton through the FAK/AKT/p70S6K/PAK1 signaling pathway and the modulation of 1433S and MTUS2 expression 32,51. However, whether different bioactive compounds, such as erinacine S share the same anticancer properties against chemoresistant CRC and elicit similar signaling activation patterns, remains uncertain. Specifically, our investigation reveals that the activation of the PAK/FAK/p300 pathways is vital in erinacine S-induced apoptosis and cell cycle arrest in HCT-116/FUR cells (Fig. 9). The PAK/FAK/p300 pathway, within a defined timeframe, is directly implicated in the initiation of oxidative stress induced by phytochemicals (Fig. 9). This highlights the redox-sensitive nature of the pathway, ultimately culminating in apoptosis 52.

Many dietary phytochemicals, commonly found in natural products and herbs, have been extensively examined for their potential to influence the pathways associated with death receptors, thereby facilitating apoptosis in cancer cells. The extrinsic pathway, involving the interaction of death activators with cell surface receptors, serves as a key mechanism for initiating programmed cell death. Notable death receptors in this context include TNF-α/Fas ligand, TNF, FasL (Apo-1L or CD95L), TNFR-1, Fas, DR4, and DR5, activated through the death region of these receptors 23-25. Often, promoting the heightened expression of death receptors and the induction of apoptosis are sought-after objectives in cancer therapy. Such efforts can effectively result in the removal of cancer cells from the cell cycle. Recent research has unveiled the involvement of the PAK/FAK/p300 pathways in apoptotic induction in CRC cells 28,33,36,37. It has become evident that the correlation mechanisms between TRAIL receptors, TNF/TNFR, and DR5, with histone modification, particularly acetylation of H3 Lys K9/K14, represent critical events in apoptosis induction through the upregulation of these death-receptor-related proteins 39,53-55. Our investigation delved into the molecular underpinnings of how erinacine S elicits apoptosis through the PAK/FAK/p300 pathway, ultimately mediating epigenetic histone H3K9K14ac of TRAIL, TNFR1, and DR5 in chemoresistant CRC cells, specifically HCT-116/FUR (Fig. 10). The emphasis on histone modifications, particularly the epigenetic aspects associated with the aggressive nature of cancer cells, is crucial in understanding how chromatin accessibility and gene expression are regulated. Further studies are imperative to comprehensively elucidate both *in vitro* and *in vivo*, how erinacine S modulates these epigenetic modifications, potentially involving histone methyltransferases (HMTs) or demethylases (HDMs) 23,56.

## Conclusions

**Figure 11:**
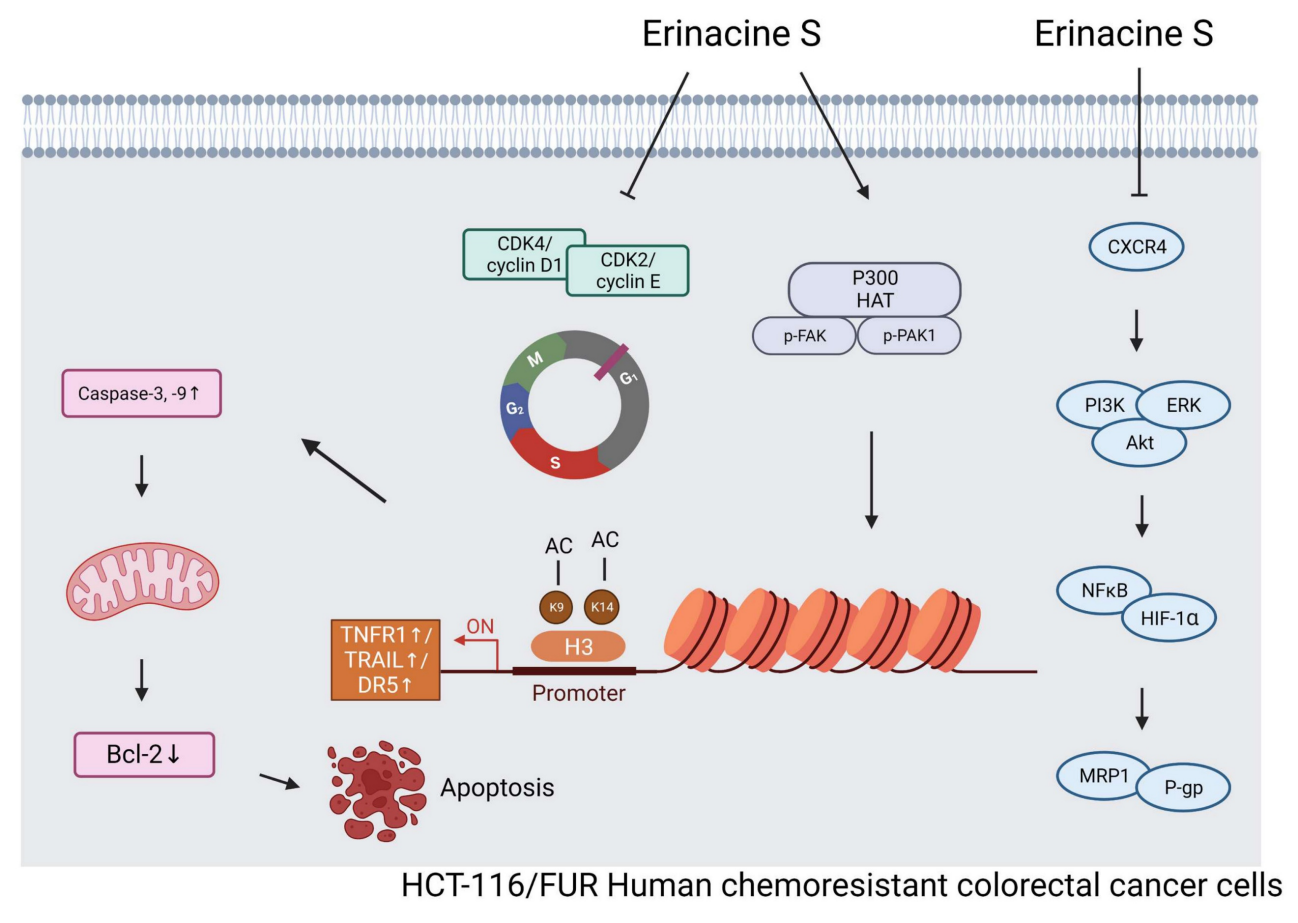
A schematic illustration of the molecular mechanism of erinacine S-mediated inhibition of proliferation, aggressiveness and cell cycle of chemoresistant human colorectal cancer HCT-116/5FUR cells. Erinacine S treatment regulated histone H3K9K14ac of the TRAIL, TNFR1, and DR5 promoters through multiple PAK/FAK/p300 pathways, resulting in the elevation of their protein level. Subsequently, the activation of TNFR1 and TRAIL by erinacine S was critical for an apoptotic pathway to either activate or downregulate caspase-3 and 9, or to inhibit the Bcl-2 protein. On the other hand, the repression of CXCR4/PI3K/AKT/ERK and NFκB/HIF1α/P-gp signaling pathways by the erinacine S treatment may lead to the repression of chemoresistant of HCT-116/5FUR cells.

In summary, this study successfully identified and characterized a drug-resistant CRC cell line, HCT-116/5FUR. It also investigated the effects of the newly extracted compound, *H. erinaceus* erinacine S, on these chemoresistant human CRC cells, highlighting its potential mechanisms of action and epigenetic regulatory properties. Importantly, erinacine S exhibited significant inhibitory effects on tumor growth in an *in vivo* xenograft mouse model. Erinacine S treatment also significantly upregulated the expression of TRAIL, TNFR1, and DR5 proteins, concurrently downregulating the CXCR4/PI3K/AKT/ERK and NFκB/HIF1α/P-gp signaling pathways in chemoresistant human CRC cells. In line with these findings, treatment of HCT-116/FUR cells with erinacine S not only activated extrinsic apoptosis pathways (TRAIL, TNFR1, DR5, and caspase 3, -9) but also suppressed the expression of anti-apoptotic molecules like Bcl-2. This process was intricately associated with the activation of the PAK/FAK/p300 pathways, and implicated the involvement of histone H3K9K14ac (acetyl Lys9/Lys14) on the promoters' regions of TRAIL, TNFR1, and DR5 (Fig. 11).

## Figures and Tables

**Table 1:** The specific primers.

TRAIL -226 to -106 bp	
TRAILf	5'-TGCATGGATCCTGA GGGCAAGG -3'
TRAILr	5'-TTGAACCTGCAACTGTCCCTCCC-3'
DR5 -199 to -75 bp	
DR5f	5'-GCCAGGGCGAAGGTTA-3'
DR5r	5' -GGGCATCGTCGGTGTAT-3'
FAS -2026 to -1849 bp	
FASf	5'-TTGGGTAACTTTGGGTGGTCC-3'
FASr	5'-ATGTGGTTGGTTGTGAAGGGAG-3'
FasL -419 to -282 bp	
FasLf	5' - GGGGGCAGTGTTCAATCTTA-3'
FasLr	5' - TGGAAAGAATCCCAAAGTGC-3'
TNFR -111 to 83 bp	
TNFRf	5' -GAT TGG TGG GTT GGG GGC ACA-3'
TNFRr	5' -ATT AAA GCA GAG AGG AGG GGA GAG A-3'
TNF-α -866 to -651 bp	
TNF-αf	5' -CAA GCA TTA TGA GTC TCC GG-3'
TNF-αr	5' -AAG CTG TGT TGA GTC CTG AG-3'

**Table 2:** Effects of the kinase inhibitors in blocking erinacine S -induced cell death in HCT-116/FUR human colorectal cancer cells.

	Apoptosis (%)
HCT-116/FUR	4
Erinacine S	28 ± 4
Erinacine S + IPA-3	5 ± 3
Erinacine S + Y15	7 ± 3
Erinacine S + C646	5 ± 3

## Abbreviations

5FUR: 5-fluorouracil-resistant
Akt: Protein kinase B
Bcl-2: B-cell lymphoma 2
Bcl-XL: Bcl-extra-large
CXCR4: C-X-C Motif Chemokine Receptor 4
CRC: Colorectal cancer
CSCs: Cancer stem cells
DR5: Death receptor 5
EMT: Epithelial to mesenchymal transition
ERK: extracellular signal-regulated kinase
FAK: Focal adhesion kinase
FOLFOX: Fluorouracil and oxaliplatin
MIF: migration inhibitory factor
PI3K: Phosphoinositide 3-kinases
